# Development of a Combined Solution Formulation of Atropine Sulfate and Obidoxime Chloride for Autoinjector and Evaluation of Its Stability

**Published:** 2013

**Authors:** Hossein Ali Ettehadi, Rouhollah Ghalandari, Alireza Shafaati, Seyed Mohsen Foroutan

**Affiliations:** a *Department of Chemical Casualties Research Center of Baghiatallah Medical Sciences University, Tehran, Iran.*; b *School of Pharmacy, Shahid Beheshti University of Medical Sciences, Tehran, Iran.*; c * Pharmaceutical Sciences Research Center, Shahid Beheshti University of Medical Sciences, Tehran, Iran.*

**Keywords:** Atropine, Obidoxime, Injectable solution, Stability test

## Abstract

Atropine (AT) and oximes, alone or in combination, have been proven greatly valuable therapeutics in the treatment of organophosphates intoxications. An injectable mixture of AT and obidoxime (OB) was formulated for the administration by automatic self-injector. The aqueous single dose solution contained 275 mg obidoxime chloride and 2.5 mg atropine sulfate per 1 mL (220 mg and 2 mg per 0.8 effective dose, respectively). The final solution was sterilized by filtration through a 0.22 μm pore size filter. This more concentrated solution allowed to use a smaller size and lighter weight cartridge. Quality control tests, including assay of the two major compounds were performed separately, using reversed-phase HPLC methods. Besides, the stability test was carried out according to ICH guideline for the accelerated test. The obtained results showed that the proposed formulation is stable over a period of 2 years after preparation.

## Introduction

Organophosphates (OPs) are widely used as insecticide in agriculture to protect livestock, crops, homes and communities from the direct and indirect effects of insects and the diseases they carry (1). Common OPs are parathion, malathion, chlorpyrifos and dichlorvos. But these compounds convey also great danger. Deliberate self poisoning has reached epidemic proportions in parts of the developing world, where the toxicity of available poisons and sparse medical facilities ensure a high fatality rate (2). Fatality rates of 20% are common, and the WHO has estimated that 200000 people die each year from pesticide poisoning (3). Organophosphonate compounds exist in a military setting as well. OP-based nerve gas was introduced in several military conflicts and acts of terror in the last few decades common names are sarin, tabun, soman and VX (3). 

**Figure 1:**
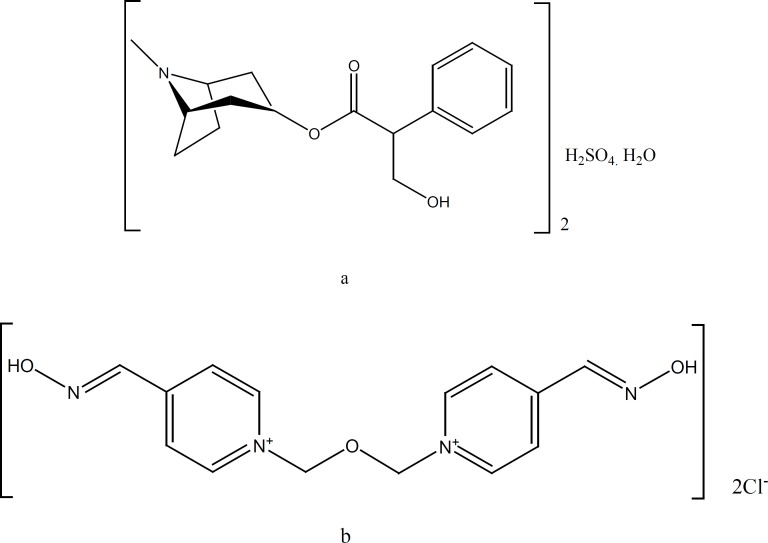
Chemical structures of atropine sulfate (a) and obidoxime chloride (b).

OP pesticides inhibit acetylcholinesterase (AChE) at muscarinic and nicotinic synapses by depositing a phosphoryl group at the enzyme›s active site. This results in an accumulation of acetylcholine and uncontrolled activation of cholinergic synapses. Standard therapy involves attempts to reduce absorption with gastric lavage and/or activated charcoal, plus administration of AT and an oxime to counter the effects of absorbed pesticide (8–10). In clinical studies, oximes in combination with AT have proven their therapeutic value in treatment of organophosphate intoxications (3, 4). In case of intoxication, a rapid self-administration of the antidotes is necessary to overcome lethal symptoms. For this reason, in the armed forces of the countries like the United States, a mixture of AT (AT) and obidoxime (OB) (Figure 1) have been used for administration by automatic injector (5-7). The concentrations of active ingredients are 110 mg OB chloride and 1.2 mg AT sulfate per one mL. In this study, we designed a more concentrated mixture of 275 mg OB chloride and 2.5 mg AT sulfate per one mL of the solution. This resulted to smaller size and lighter weight autoinjector, which is easier to carry by individuals. 

## Experimental

*Materials*

AT sulfate was purchased from Boehringer- Ingelheim Company (Ingelheim, Germany). OB chloride, phenol, hydrochloric acid, tetramethyl ammonium chloride, analytical-grade 1-octane sulfonic acid sodium salt and HPLC-grade acetonitrile were obtained from Merck Chemical Co. (Darmstadt, Germany) 

Glass dental cartridges of USP type I was purchased from Daroushisheh Co (Tehran, Iran). 

*HPLC system*

All assays were carried out using a Knauer HPLC system (Germany) consisted of a model Wellchrom K-1001 pump, a model Rheodyne 7125 injector and a model K 2501 UV detector controlled by Eurochrom 2000 (Integration Package) software. 

*Chromatographic conditions*

The separation was performed on an analytical 25 × 4.6 mm i.d*. *MZ C18 (5 μm, particle size) column. The mobile phase was a mixture of acetonitrile and phosphate buffer 50 mM (13:87 v/v, respectively) containing 1 mM octanesulfonic acid and 5 mM trimethylammonium chloride adjusted to pH of 3.5 at a flow rate of 1 mL/min. The mobile phase was freshly prepared daily and degassed by ultrasonification before use. The mobile phase was prepared freshly and was filtered before use. Column was maintained at ambient temperature. The detector was set at 220 nm. 

*Cartridge preparation*

Steam-sterilized glass cartridge, convoluted needle and the septums were placed and aligned. Then, in a phenolic aqueous solution (0.5% w/v), OB chloride (275 mg/mL) and AT sulfate (2.5 mg/mL) were dissolved and the pH of the solution was set at 3-3.5 by adding few drops of 1M HCl solution. The solution was filtered through a 0.22 μm membrane filters(milipore Corps.Mass, USA). Each dental glass cartridges was filled with 0.9 mL of the solution, covered with the front septum under nitrogen gas flow and sealed with an aluminium cap using the hand operated crimping device. Three series of cartridges were prepared in different dates at 2 month intervals, while each series consisted 3 batches of 10 cartridges. 

*Preparation of standard and assay solutions*

Twenty mg of each of AT sulfate, OB chloride and phenol were weighed and transferred to 20 mL volumetric flask. Then, diluent mixture containing acetonitrile-phosphate buffer (20:80, v/v) was added to the volume. The working solutions were prepared from the stock solutions by adding diluents solution. 

The content of autoinjector cartridge containing a phenolic aqueous solution (0.5% w/v), OB chloride (275 mg/mL) and AT sulfate (2.5 mg/mL) was released in 5 mL water. This solution was diluted to 25 mL with the diluents mixture. Then, 1 mL of this solution was again transferred to 10 mL volumetric flask and diluted with the diluents mixture to obtain 1100 μg/mL of OB and 10 μg/mL of AT. 

*Assay and stability test: *The stability study was conducted according to accelerated stability test protocol recommended by ICH (14). Sufficient number of samples were taken from three pilot batches and stored at 40ºC temperature and 75% relative humidity. The active ingredients of the solutions were assayed using the HPLC method developed specifically for measuring at the time of preparation and at 2 month intervals up to 6 months. 

## Results and Discussion

*Autoinjector device*

**Figure 2:**
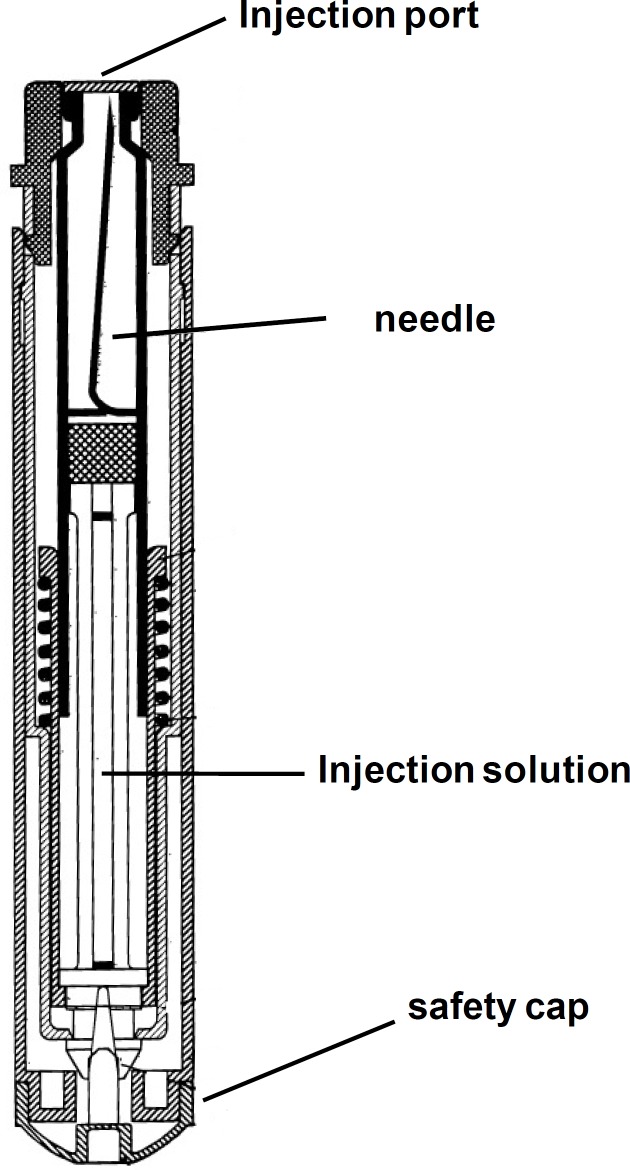
A schematic diagram of the autoinjector device with the cartridge inside containing mixed solution of atropine and obidoxime.

A schematic of the device is shown in Figure 2. 

The dental cartridge containing 0.9 mL of the mixed solution was installed inside the device. By pressing the injection port on, for example thigh muscle, the needle automatically inserted into the muscle and releases the solution in the muscle. In each injection, the device effectively delivers 0.8 mL of the solution to the body, which contained 220 mg obidoxime chloride and 2 mg atropine sulfate per dose. 

*Development of assay method*

**Figure 3:**
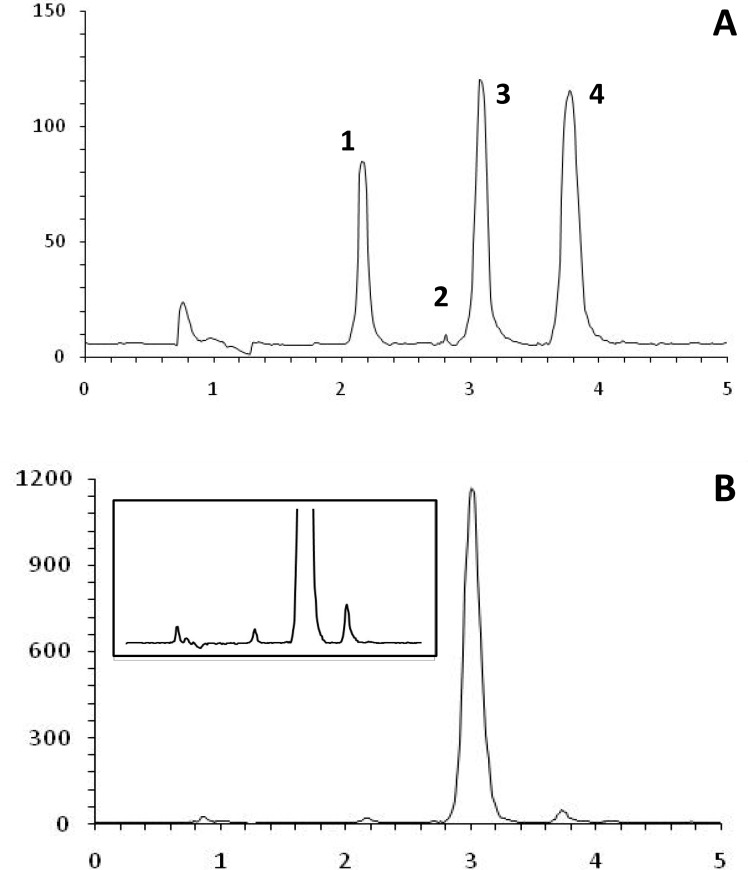
Chromatograms showing (A) separation of AT sulfate (1), tropic acid (2), obidoxime (3) and phenol in a standard solution containing 0.05 mg/mL of each compound, and (B) separation of components of a cartridge.

The proposed HPLC method was developed specifically for assay of the injection solution, which contains two main components, AT and OB, as well as phenol as preservative. Reported HPLC methods differ with respect to the mode of detection (ultraviolet or electrochemical) and sample preparation (solvent extraction and solid-phase extraction) (15). Thus, the key response in method development was to resolve three components of the injection solution at one single run. Figure 3 shows the chromatogram obtained at the optimal HPLC conditions. Baseline separation of the cartridge components was achieved in less than 5 min. The method selectively allows measuring each component in the solution. Addition of trimethylammonium chloride to the mobile phase resulted in sharp peaks, due to elimination of possible interaction of AT and OB with the stationary phase. At the same time, presence of octanesulfonic acid improved retention time of the both basic components by making temporary ion-pair complexes with AB and OB. 

*Validation of the method*

The method was validated according to the International Conference for Harmonization (ICH) guidelines (16). The selectivity of the method was assessed by measuring resolution between each pair of adjacent peaks. The response for the detector was determined to be linear over the range of 5-60 μg/mL for AT, 50-1200 μg/mL for OB and 10-100 μg/mL for phenol. Each of the concentration was injected in triplicate to get reproducible response. Correlation coefficients (*r*) for the 3 compounds were ≥ 0.998. The linear regression equation was *y *= 270*x *+ 455 for AT and *y *= 1037*x *+ 1739 for OB, where *y *is the peak area and *x *is the concentration in μg/mL. 

Precision of the method was examined as intra- and inter-day variation measurements at 3 concentration levels of AT (2, 8 and 20 μg/ mL) and OB (200, 400 and 800). Six replicate quality control samples at each concentration were assayed on the same day for intra-day variation assessment. The inter-day precision was evaluated on three different days. The R.S.D. values were found to be 1.4% for AT and 0.7% for OB indicating acceptable repeatability. The R.S.D. values according to the retention time at the same day at each concentration level changed between 0.1% and 1.6%, indicating again very good repeatability.

Accuracy was determined by adding known amounts of each drug at concentrations of 5, 10 and 15 μg/mL for AT and 60, 140 and 200 μg/mL for OB to the excipients and measuring percent recovery of the drug from each sample in triplicate. The average recovery was 96.3 ± 1.2% for AT and 101.1 ± 0.8% for OB, respectively.

*Stability Studies*

Table 1 shows the results of analysis of OB samples obtained from cartridges at the defined intervals, compared to the stability of control sample solutions of OB (see Table 2). The results shown for each batch are the average of 3 samples analyzed. It is necessary to mention that the acceptance limits of concentration are 90%-110%.

**Table 1:** Results of stability studies of the proposed formulation shown as percentage of OB in 3 batches (N = 3, n = 3) at 2 month intervals.

**Date of Sampling**	**BatchA**	**BatchB**	**BatchC**	**Mean ± SD**
**0**	101.3	96.3	96.3	97.2 ± 2.9
**2** ^nd^ **month**	92.1	100.7	96.5	96.4 ± 4.3
**4** ^th^ **month**	98.9	95.9	95.1	96.6 ± 2
**6** ^th^ **month**	99.3	100.1	96.5	98.6 ± 1.9

**Table 2:** Results of stability studies of the proposed formulation shown as percentage of AT in 3 batches (N = 3, n = 3) at 2 month intervals.

**Date of Sampling**	BatchA	BatchB	BatchC	Mean ± SD
0	107.3	103.2	102.6	104.3 ± 2.8
2^nd^ month	99.8	106.1	103.3	103.1 ± 3.2
4^th^ month	103.3	101.1	104.4	102.9 ± 1.7
6^th^ month	105.1	102.3	102.6	103.3 ± 1.5

## Conclusion

In this paper, preparation of a mixed solution of atropine and obidoxime for an autoinjector device was described. Furthermore, the HPLC method specifically developed for the assay of two components in the solution was proved to be robust and reliable. The method was successfully applied for the assay and stability study of the solution formulation. 
